# On the Contagion of Inoculated Small-Pox

**Published:** 1811-09

**Authors:** 


### *For the Medical and Physical Journal.*

### *On the Contagion of Inoculated Small-Pox.*

(Continued from page 120.)

I AM perfectly in accord with you, gentlemen, that this is an "interesting," I will say, a very momentous "inquiry." I will therefore prove, with your permission, that the other assertion of Dr. Douglass, which attributes the excessive and rapid propagation of the natural small-pox through the town of Boston to inoculation, is not so void of probability as to be considered of "but little value."

In support of this assertion, Dr. Douglass compares the progress and mortality of the last small-pox, with that which had occurred about nineteen years before*.

1st. He tells us, "that in the former small-pox season, nineteen years before, the disease spread gradually; that few, comparatively, were infected at a time; that its progress occupied a considerable space of time, *viz.* from ten to twelve months; that vast numbers escaped the infection altogether; and that the total number of deaths throughout this long period, did not amount to *four hundred.*"

2dly. He informs us, "that the mortality from the small-pox was greatest in the month of December, during which month, *eighty* persons died of the disease."

I do not find, that the accuracy of this statement has been disputed; and when we consider the extreme dread of the small-pox, which was then universal among all ranks of people, and the very great care which was taken to guard against infection, it seems highly probable, that the progress of the disease would be as slow as is here described.

If the number of those who died was mistated, we should surely have found the statement contradicted. It would be the obvious policy of the Doctor's opponents to prove, that the virulence and mortality of the small-pox were as great before inoculation was introduced as afterwards. It is hardly possible that the Rev. Mr. Mather, the great advocate of

---

part from their established principle of conduct, with the hope that the gentleman, at whose country residence the case occurred, will confirm the fact. Perhaps accident may some time discover a remedy for this dreadful malady, when theory, science, and reasoning, have failed.

* It can hardly be necessary to inform any of your readers, that the total absence of variolous contagion from particular districts, for a long series of years, was formerly no unusual circumstance.

inoculation inoculation at Boston, should not have had the means of ascertaining this point; and he, who had been so rudely treated by Dr. Douglass, would, doubtless, have been well disposed to disprove the Doctor's assertions on this head, had the assertions been disputable.

Thus much for the first season of small-pox: of the second, which was on the decline, or nearly worn out, when Douglass wrote, we learn the following particulars.

1st. That the diffusion of the infection was much more general, and the progress more rapid: for, in the *three months of September, October, and November*, they buried *seven hundred and sixty persons, which is nearly twice as many as died of the small-pox, in the whole period of ten or twelve months*, nineteen years before.

2dly, That the mortality was greatest in the month of October, during which month, *upwards of four hundred persons* died, which is *five times as many* as died of the same disease, during the month of the greatest mortality in the former season.

It may be collected from the testimony of the Rev. Mr. Mather, that the progress of the disease and the number of deaths, as given in this statement, was tolerably correct; for he tells us* that, "in little more than half a year, *upwards of five thousand persons* underwent the small-pox," and "near nine hundred died of it:" and he does not contradict what is said of the great mortality in the months of September, October, and November.

Mr. Mather likewise acknowledges, that during the prevalence of this epidemic, "*almost three hundred persons*" were inoculated; the first inoculation being undertaken towards the latter end of June, when the natural small-pox had prevailed about two months: for it commenced at Boston in April. It is evident, therefore, that the rapid increase of the pestilence was coincident with the practice of inoculation.

The question then to be resolved is this. Is the promiscuous inoculation of the small-pox, as a cause, equal to the diffusion of the infection as an effect?

That the inoculation of the small-pox at Boston was equal to produce this effect, is, I think, undeniable; when it is considered that the inoculators unwittingly taught, *that the small-pox by inoculation was not infectious*, and when, of course, little or no care was taken to guard against the infection; or indeed, when, from the promulgation of such an

† Rev. Mr. Mather's Letter, dated March 10, 172½, in "Maitland's Account of Inoculating the Small-Pox vindicated." p. 58.

opinion, opinion, people were rather courted to run into the way of danger.

If, from *one* child inoculated by Mr. Maitland, the infection spread to *six* persons and *one* died;—

If, from the inoculations of *one* man* who taught his patients, "that the small-pox was not catching from the inoculated," *the whole neighbourhood* became infected and *several* died;—

If, from *one* child inoculated, as we are told, by Dr. Willan†, *seventeen* persons took the infection and *eight* died;—

What reason is there to doubt that an excessive diffusion of variolous infection, and consequent mortality, would be created from the inoculation of "almost three hundred persons," at Boston, where no precautions were adopted to separate the inoculated from those liable to be infected?

Does Mr. Mather deny that inoculation tended to spread the disease? I cannot find that he does; but he urges the necessity of inoculation, because, of the numbers inoculated none, he says, died *of* the disease, and only five or six *upon* it, or *after* it‡.

Does Mr. Maitland deny the infectious nature of the inoculated small-pox? We have already seen what he calls "a *full* answer to this objection;" *valeat quantum valere potest*—but he tells us, that "it would be a most tyrannical encroachment upon the natural rights of mankind, to debar them from the lawful means of securing themselves from the fear and danger of so terrible a plague.§" A sufficient acknowledgement, that considerable apprehensions were entertained by others, of the diffusion of infection from the inoculated small-pox.

As a matter of fact, it has been ascertained, "from a comparison of the Bills of Mortality for a series of years in various places, that a larger proportion of the inhabitants has died of the small-pox, in towns where inoculation is practised, than in the same before it was known, or in others where it is prohibited.||"

I have now, I presume, demonstrated, that there is much appearance of truth and consistency in Dr. Douglass's assertions; I hope, therefore, it will be conceded to me, that I

* Baron Dimsdale's Thoughts on General and Partial Inoculations, p. 10, 1776.

† Reports on Diseases of London, p. 18. 1801.

‡ "Maitland's Account of Small-Pox vindicated. P. 58.

§ Ibid. p. 34.

|| Haygarth's Sketch of a Plan to exterminate the casual Small-Pox. 29, 1793.

have not greatly erred in appealing to his testimony, expressed in very strong, if not in very polite language, in support of the argument, that the infection of the natural small-pox may arise from inoculation. A conviction of the truth of this fact has drawn from the governors of the Small-pox Hospital a peremptory prohibition of *indiscriminate* inoculation, and I sincerely hope, that their example may be followed by the governors of all other institutions, where the promiscuous inoculation of the small-pox is still permitted and practised.

I am sensible, Gentlemen, that I have occupied in this discussion more space than ought, perhaps, to be afforded to an anonymous correspondent; if, however, there is as much danger as I imagine, in having it believed that the inoculated small-pox is *not infectious*, the pages of your Journal will not have been employed to a useless purpose, in pointing out that danger.

As the facts referred to have been long before the public, and as nothing in this letter is advanced solely upon the responsibility of the writer, he still prefers concealing his real name, under the signature

REPREHENSOR.

*July 8th*, 1811.

